# Interleukin-18 and Hematopoietic Recovery after Allogeneic Stem Cell Transplantation

**DOI:** 10.3390/cancers12102789

**Published:** 2020-09-28

**Authors:** Aleksandar Radujkovic, Lambros Kordelas, Rashit Bogdanov, Carsten Müller-Tidow, Dietrich W. Beelen, Peter Dreger, Thomas Luft

**Affiliations:** 1 Department of Internal Medicine V, University Hospital Heidelberg, 69120 Heidelberg, Germany; Carsten.Mueller-Tidow@med.uni-heidelberg.de (C.M.-T.); Peter.Dreger@med.uni-heidelberg.de (P.D.); Thomas.Luft@med.uni-heidelberg.de (T.L.); 2 Department of Bone Marrow Transplantation, University Hospital Essen, University of Duisburg-Essen, 45147 Essen, Germany; Lambros.Kordelas@uk-essen.de (L.K.); Rashit.Bogdanov@uk-essen.de (R.B.); Dietrich.Beelen@uk-essen.de (D.W.B.)

**Keywords:** interleukin-18, hematopoietic recovery, allogeneic stem cell transplantation, non-relapse mortality

## Abstract

**Simple Summary:**

We have previously shown that high pre-conditioning levels of Interleukin-18 were associated with worse survival after allogeneic stem cell transplantation due to increased non-relapse mortality. While no correlations with acute graft-versus-host disease were observed, interleukin-18-related excess mortality was mainly driven by fatal infectious complications. In multiple studies, delayed hematopoietic recovery and poor graft function following allogeneic stem cell transplantation has been demonstrated as a powerful predictor of non-relapse mortality. The present study links high interleukin-18 to delayed platelet recovery in allografted patients. Given the functions of interleukin-18 in regulating the quiescence of hematopoietic stem/progenitor cells, our findings may be explained by Interferon gamma-independent inhibitory effects of interleukin-18 on stem cell proliferation and hematopoietic reconstitution in allografted patients. Importantly, considering recent successful interleukin-18-neutralizing approaches in autoimmune disorders, our results provide a rationale to explore modulation of interleukin-18 for improving hematopoietic recovery and outcomes in allogeneic stem cell transplantation recipients.

**Abstract:**

Interleukin-18 (IL-18) is an immunoregulatory cytokine and a context-dependent regulator of hematopoietic stem/progenitor cell (HSPC) quiescence in murine models. In a previous study, high pre-conditioning levels of IL-18 were associated with increased non-relapse mortality (NRM) after allogeneic stem cell transplantation (alloSCT). To investigate the clinical impact of IL-18 status on hematopoietic function, the associations of pre-conditioning and day 0–3 cytokine levels with platelet and neutrophil recovery were analyzed in a training cohort of 714 allografted patients. In adjusted logistic regression analyses, both increasing pre-conditioning and day 0–3 IL-18 levels had a significantly higher adjusted odds ratio (aOR) of delayed platelet and neutrophil recovery on day +28 post-transplant (aOR per two-fold increase: 1.6–2.0). The adverse impact of high pre-conditioning IL-18 on day +28 platelet recovery was verified in an independent cohort of 673 allografted patients (aOR per two-fold increase: 1.8 and 1.7 for total and free IL-18, respectively). In both cohorts, a platelet count ≤20/nL on day +28 was associated with a significantly increased hazard of NRM (hazard ratio 2.13 and 2.94, respectively). Our findings support the hypothesis that elevated peritransplant IL-18 levels affect post-transplant HSPC function and may provide a rationale to explore modulation of IL-18 for improving alloSCT outcomes.

## 1. Introduction

Allogeneic stem cell transplantation (alloSCT) is an established curative approach for a wide array of malignant and non-malignant hematologic disorders. However, besides persistence or recurrence of the primary disease, several complications can occur, including hemorrhages, infections, graft-versus-host disease (GVHD), and endothelial damage-related toxicities, translating into considerable treatment-associated morbidity and mortality.

Robust recovery and reconstitution of a donor-derived hematopoietic system in the recipient is of key importance for successful alloSCT. Accordingly, delayed hematopoietic recovery and poor graft function are associated with unfavorable outcomes [1,2,3,4]. In particular, delayed platelet recovery has been demonstrated to be a powerful predictor of increased non-relapse mortality (NRM) [2,5,6,7,8,9].

Interleukin-18 (IL-18) is a pro-inflammatory, interferon (IFN)γ-inducing, immunoregulatory cytokine involved in both innate and adaptive immune responses [10,11,12]. The IL-18 precursor is constitutively expressed in nearly all cells in humans including endothelial cells [10]. Maturation and secretion of IL-18 is mediated by the inflammasome [12], and its activity is inhibited by high-affinity binding with IL-18 binding protein (IL-18BP) [10,11].

In clinical settings, increased levels of IL-18 have been associated with a variety of autoimmune disorders including GVHD [11,13]. In addition, being part of the human host defense against infection, elevated IL-18 was reported to correlate with infection severity and outcome [14,15]. Accordingly, higher pre-conditioning levels of free IL-18 were found to be associated with non-relapse and overall mortality after alloSCT [16]. While no correlations with acute GVHD were observed, IL-18-related excess mortality was mainly driven by fatal infectious complications.

Using proximity-based differential single-cell analysis of the bone marrow (BM) niche Silberstein et al. [17] identified a previously unrecognized function of IL-18 as a regulator of hematopoietic stem/progenitor cells (HSPC) quiescence. In murine transplant models, IL-18 protected HSPC from injury caused by exposure to the cell-cycle-specific genotoxin 5-fluorouracil (5-FU), but in doing so, limited HSPC proliferation. In the clinical context of alloSCT, these findings may suggest a role of IL-18 in regulating post-transplant hematopoietic recovery and homeostasis.

IL-18 induces IFNγ, and IFNγ conversely increases the gene expression and synthesis of both IL-18BP and several IFNγ-inducible CXC family chemokines. However, it should be noted that the BM niche factor function of IL-18 regulating HSPC quiescence is likely to involve IFNγ-independent effects of IL-18 [17], and therapeutic modulation of the IL-18 pathway is considered to be more effective in conditions with a minor role for IFNγ [18].

In light of the above considerations, in the present study, we sought to investigate a possible link between IL-18 status and delayed post-transplant hematopoietic reconstitution focusing on IL-18, IL-18BP, IFNγ and the IFNγ-inducible CXC family chemokines CXCL9 and CXCL10.

## 2. Results

### 2.1. Patient Characteristics and Cytokine Serum Levels

Patient characteristics of both cohorts are summarized in Table 1. There were more patients with late stage and lymphoid disease in the training cohort. In the confirmation cohort, fewer patients had received reduced intensity conditioning, ATG in-vivo T-cell depletion and mycophenolate mofetil-containing GVHD prophylaxis. Baseline cytokine levels of both cohorts are also given in Table 1. Levels of total IL-18 and IL-18BP were higher in the confirmation cohort, whereas levels of free IL-18 were similar in both cohorts.

Demographics, disease and transplant characteristics of the subgroup of patients who had serum samples available at day 0–3 peri-transplant (*n* = 306) were comparable to that of the entire training cohort Table S1. Pre-conditioning serum levels of total IL-18, IL-18BP as well as free IL-18 were lower than the levels observed at day 0–3 peri-transplant. Levels of IL-18 levels (total and free) were positively correlated (Table S2).

Pre-conditioning and day 0–3 IL-18 levels were not associated with patient age, patient gender, the hematopoietic cell transplantation-specific comorbidity index (HCT-CI) and the disease stage prior to alloSCT in the training cohort. Pre-conditioning total and free IL-18 levels were increased in patients with higher C-reactive protein (CRP) and ferritin levels prior to the start of the conditioning. For day 0–3 total and free IL-18 levels only a trend towards increased levels in patients with higher pre-conditioning CRP levels was observed, whereas pre-conditioning ferritin levels were not associated with day 0–3 IL-18 (Figures S1–S4).

### 2.2. Platelets and ANC in Patients with Low Versus High IL-18 Levels

**Figure 1:**
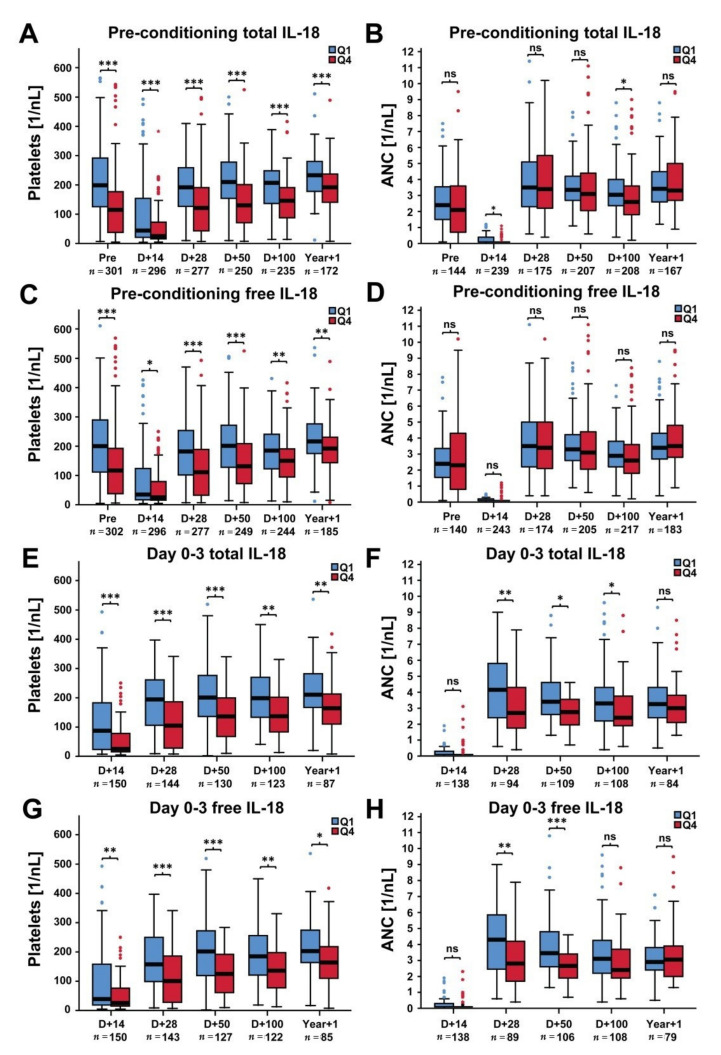
Median platelet and neutrophil counts pre-conditioning and at different time-points following alloSCT in patients with low versus high pre-conditioning and day 0–3 total and free IL-18 levels (training cohort). (**A**,**B**) Median platelet and neutrophil counts, respectively, in patients with low versus high pre-conditioning total IL-18 levels. (**C**,**D**) Median platelet and neutrophil counts, respectively, according to pre-conditioning free IL-18 levels. (**E**,**F**) Median platelet and neutrophil counts, respectively, in patients with low versus high day 0–3 total IL-18 levels. (**G**,**H**) Median platelet and neutrophil counts, respectively, according to day 0–3 free IL-18 levels. The first (Q1) and fourth quartile (Q4) of the total IL-18 and free IL-18 distribution was used to stratify patients in low and high IL-18 groups. Corresponding patient numbers are given (*n* = Q1 + Q4). Mann–Whitney U test, * *p* < 0.05, ** *p* < 0.01, *** *p* < 0.001, ns = not significant.

Platelet and neutrophil counts were evaluated with regard to both total and free IL-18 in the present study. High levels of pre-conditioning total and free IL-18 (as defined by the fourth quartile (Q4) of the corresponding distribution) were associated with significantly lower median platelet counts as compared to low IL-18 levels (Q1) at all time-points investigated including the pre-conditioning landmark (Figure 1A,C). The differences in median platelet counts in patients with low (Q1) versus high (Q4) pre-conditioning IL-18 levels diminished with increasing time from transplant. No significant associations of IL-18 levels with regard to ANC were observed (Figure 1B,D).

Similar associations were observed for day 0–3 total and free IL-18 and platelet counts (Figure 1E,G). In addition, high day 0–3 IL-18 levels were also correlated to lower median neutrophil counts at day +28 and day +50 post-transplant (Figure 1F,H).

**Figure 2:**
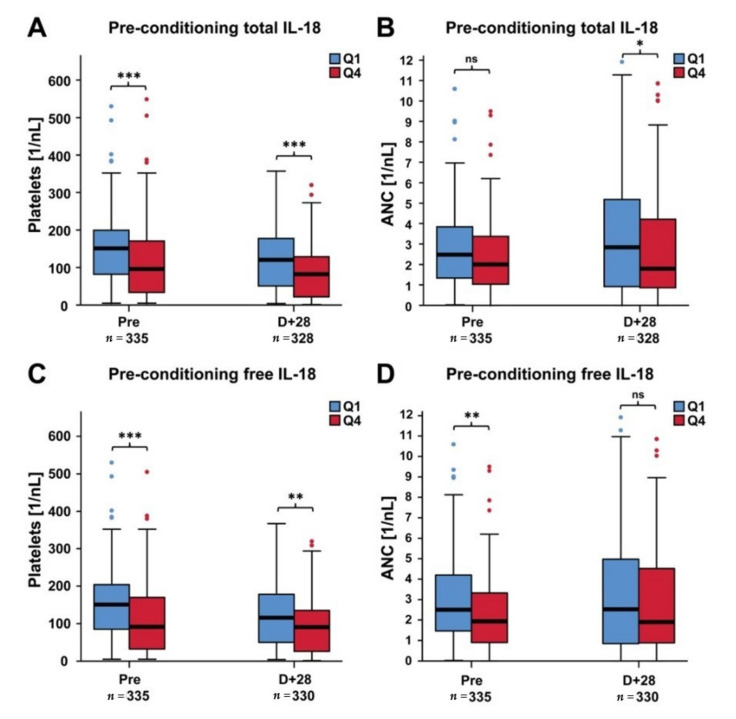
Median platelet and neutrophil counts pre-conditioning and at day +28 following alloSCT in patients with low versus high pre-conditioning total and free IL-18 levels (confirmation cohort). (**A**,**B**) Median platelet and neutrophil counts, respectively, in patients with low versus high pre-conditioning total IL-18 levels. (**C**,**D**) Median platelet and neutrophil counts, respectively, according to pre-conditioning free IL-18 levels. The first (Q1) and fourth quartile (Q4) of the total IL-18 and free IL-18 distribution was used to stratify patients in low and high IL-18 groups. Corresponding patient numbers are given (*n* = Q1+Q4). Mann–Whitney U test, * *p* < 0.05, ** *p* < 0.01, *** *p* < 0.001, ns = not significant.

In the confirmation cohort, patients with high pre-conditioning levels of total and free IL-18 had significantly lower median platelet counts both before alloSCT and at day +28 post-transplant (Figure 2A,C), whereas higher levels of pre-conditioning total and free IL-18 were associated with lower median ANC on day +28 and prior to alloSCT, respectively (Figure 2B,D).

The corresponding Spearman’s rank correlation coefficients for both cohorts were summarized in Table S3.

### 2.3. Cytokine Serum Levels and Hematopoietic Recovery

In the training cohort, higher pre-conditioning total and free (continuous) IL-18 levels predicted non-achievement of defined platelet and neutrophil recovery milestones (Table 2). As per area under the ROC curve, the most informative cytokine for predicting delayed platelet recovery on day +28 was free IL-18. In contrast, no associations of platelet and neutrophil recovery with pre-conditioning levels of IL-18BP, IFNγ and the IFNγ-inducible chemokines CXCL9 and CXCL10 were observed (Table 2). For day 0–3 IL-18 levels, similar and stronger associations were found in univariate analysis particularly for day +28 platelet and neutrophil recovery on (OR ≥ 2) (Table 3). In addition, a correlation between delayed platelet recovery and higher levels of IL-18BP and IFNγ at day 0–3 was observed but only for the day +50 milestone.

In multivariable analyses adjusting for donor match, graft source, ATG use, conditioning intensity, and disease score, increasing pre-conditioning IL-18 levels (total and free) were significantly associated with missing the day +28 platelet and neutrophil milestones (adjusted OR per two-fold increase 1.6–1.8, Table 4). Multivariable models including day 0–3 IL-18 serum levels yielded similar and more pronounced associations (adjusted OR per two-fold increase 1.7–2.0, Table S4).

In the confirmation cohort, higher pre-conditioning levels of both total IL-18 and IL-18BP were associated with increased odds of delayed platelet recovery on day +28 (Table 5). For free IL-18, only a trend towards a higher OR of delayed platelet recovery was observed, whereas no associations with delayed neutrophil recovery were found in univariate analyses (Table 5).

However, after multivariable adjusting for confounders, increasing pre-conditioning levels of both total and free IL-18 correlated with non-achievement of the day +28 platelet recovery milestone (adjusted OR 1.8 and 1.7, respectively), whereas the associations with neutrophil recovery did not reach statistical significance (Table 6).

### 2.4. Day +28 Platelet Recovery and Outcome

We have previously reported that elevated pre-conditioning IL-18 is a predictor of worse survival due to increased NRM (HR per doubling in IL-18, ~1.2–1.4 in both centers) [16].

**Figure 3:**
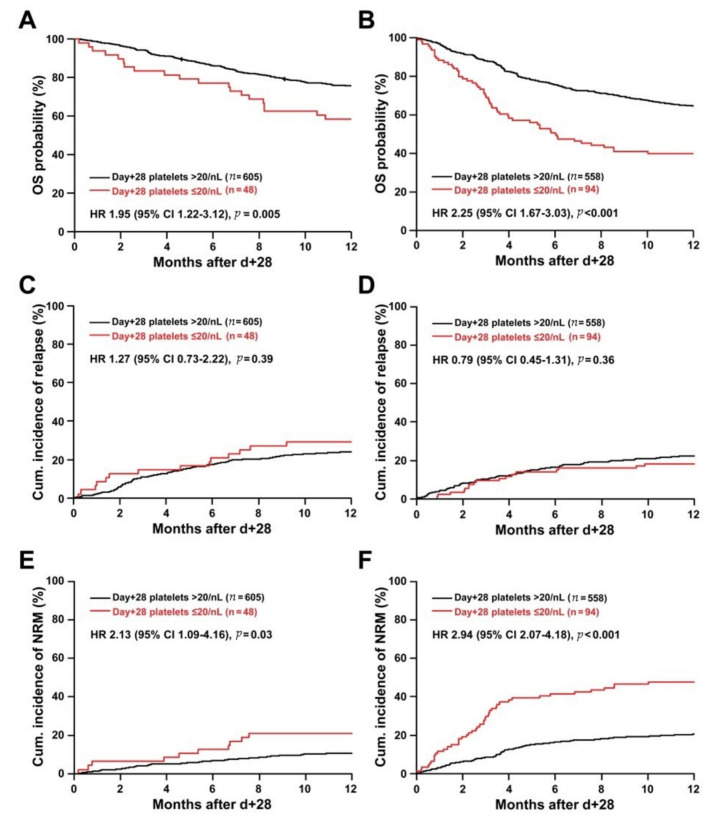
Outcome of allografted patients in the first year post-landmark stratified according to day +28 platelet counts in the training cohort and in confirmation cohort. (**A**) In the training cohort (*n* = 653), platelets ≤20/nL at day +28 were associated with lower probability of overall survival (OS) with the first year after day +28. (**C**) Platelets ≤20/nL at day +28 were not correlated with incidence of relapse in the training cohort. (**E**) In contrast, platelets ≤20/nL at day +28 were associated with a significantly increased cumulative incidence of non-relapse mortality (NRM) in the training set. (**B**,**D**,**F**) In the confirmation cohort (*n* = 652), a comparable association of platelet counts <20/nL with worse OS, due to a higher incidence of NRM rather than relapse, was observed. Note: Patients who experienced relapse within the first 28-day post-transplant were excluded from the analysis. Abbreviations: CI, confidence interval; HR, hazard ratio; OS overall survival, NRM, non-relapse mortality.

On univariate analysis, a platelet count ≤20/nL on day +28 was associated with significantly decreased OS within one year post-landmark in both cohorts (HR 1.95 95%CI 1.22–3.12, *p* = 0.005 and HR 2.25 95%CI 1.67–3.03, *p* < 0.001, respectively). This was due to markedly increased hazards of NRM (HR 2.13 95%CI 1.09–4.16, *p* = 0.03 and HR 2.94 95% CI 2.07–4.18, *p* < 0.001 in the training and the confirmation cohort, respectively), but not to relapse deaths. The results of the univariable analyses were depicted in Figure 3. The association of the non-achievement of the day +28 platelet recovery milestone with increased NRM in both cohorts could be further substantiated in the corresponding multivariable models (Table S5).

## 3. Discussion

The present study demonstrates an association of increased IL-18 serum levels with impaired hematopoietic reconstitution in alloSCT recipients. In particular, high IL-18 correlated with a delayed platelet recovery. In contrast, IFNγ, or the inflammatory IFNγ response chemokines CXCL9 and CXCL10, were not associated with hematopoietic recovery in our study. In light of preclinical evidence that IL-18 is a novel and context-dependent regulator of HSC quiescence [17], our findings may be explained by a direct inhibitory effect of IL-18 on stem cell proliferation in allografted patients.

Affecting up to 27% of patients in some studies [2], poor graft function is a relevant complication of alloSCT associated with infections and hemorrhages and considerable morbidity and mortality [3]. Both the speed and the quality of post-transplant hematopoietic recovery may be affected by various factors, including disease status at transplant, stem cell source, human leukocyte antigen (HLA) compatibility, donor type, intensity of the conditioning regimen, and the use of in vivo T-cell depletion [3]. Usually, the time to achieve a given neutrophil or platelet count is used as indicator of engraftment/recovery of the recipient’s hematopoietic system. However, this parameter does not necessarily reflect the robustness of hematopoietic recovery in the long term after alloSCT. In addition, cytopenias observed following alloSCT may be the result of both slow recovery of blood counts and decreasing blood counts after initially successful engraftment [3].

Delayed platelet recovery following alloSCT is a well-acknowledged risk factor for increased NRM and poor survival [2,5,6,7,8,9]. The underlying mechanism of prolonged thrombocytopenia after alloSCT is complex and may include both impaired thrombopoiesis and increased platelet turnover [23]. Several studies suggest reduced differentiation of megakaryocytes from stem cells and defects in megakaryocytic maturation rather than peripheral destruction of platelets to account for prolonged thrombocytopenia after alloSCT [24,25]. Recently, direct damaging effects of inflammation-associated cytokines on ex vivo megakaryocytic and hematopoietic stem cell function have been demonstrated [26].

In our study, higher levels of pre-conditioning total and free IL-18 were correlated with significantly lower median platelet counts but not ANC, both prior to the start of the conditioning and at various time-points after alloSCT. By comparison, high levels of total and free IL-18 measured at day 0–3 were associated with both lower median platelet counts up to one year after alloSCT and lower median ANC up to day +50 post-transplant. Accordingly, increasing levels of both pre-conditioning and day 0–3 total and free IL-18 were correlated with the risk of non-achievement of day +28 platelet and ANC recovery milestones. These associations were even stronger for day 0–3 IL-18 levels and were decreasing over time with respect to platelets. Interestingly, no associations of IFNγ and the IFNγ-inducible CXC family chemokines CXCL9 and CXCL10 on platelet and neutrophil recovery were observed. Since IL-18 is an IFNγ-inducing cytokine and IL-18 and CXC family chemokines show a high level of intercorrelation [16], these findings suggest that myeloid recovery might be affected by IL-18 independently of IFNγ.

In the context of alloSCT, IL-18 has been investigated primarily with respect to GVHD but clinical studies were often limited by the small numbers of subjects investigated [27,28,29,30]. Consequently, a correlation of IL-18 with GVHD severity was found in some alloSCT studies [27,28] but not in others [29,30]. We have previously reported that elevated pre-conditioning IL-18 levels predicted worse survival due to increased NRM [16]. In this previous study, median levels of total and free IL-18 in healthy controls were 175 and 141 pg/mL, respectively, which is approximately 4- and 3-fold lower as compared to the median pre-conditioning levels observed in allografted patients [16]. We can only speculate about the underlying causes. In the present study, IL-18 levels were not associated with patient age, patient gender, the HCT-CI and the disease stage prior to alloSCT. However, pre-conditioning total and free IL-18 levels were significantly higher in patients with elevated CRP and ferritin levels consistent with a pro-inflammatory state. Given the implications of the IL-18/IL-18BP pathway in endothelial dysfunction [31,32] and the fact that IL-18 may be released from dying endothelium [11], high IL-18 levels in patients undergoing alloSCT may also reflect endothelial distress caused by the underlying hematologic malignancy and/or the previous antineoplastic treatment.

IFNγ-independent effects of IL-18 have gained recent attention [18]. In murine transplant models, BM inflammation involving increased inflammasome activation and enhanced secretion of IL-18 and other cytokines has been demonstrated [33]. Consequently, inhibition of caspase-1 and inflammasome activation was shown to attenuate BM inflammation and promote hematopoietic reconstitution as reflected by increased numbers of megakaryocytes and platelets in transplanted mice. Notably, in their elaborate study on BM niche factors Silberstein and colleagues [17] demonstrated IL-18 as a previously unrecognized quiescence regulator of short-term HSPC. By limiting HSPC proliferation in murine transplant models, IL-18 was able to protect HSPC from 5-FU-induced apoptosis, however, at the expense of a constrained hematopoietic recovery. Similar observations were made by Bordoni et al. [34] in the context of primary HIV infection and the IL-18 stress-response with respect to in vivo proliferation and expansion of lymphoid progenitor stem cells. As regards our observations, the lack of correlation between platelet and neutrophil recovery and levels of IFNγ and IFNγ-inducible chemokines may suggest specific and IFNγ-independent effects of IL-18 as a regulator of HSPC quiescence in patients undergoing alloSCT. This is of particular relevance, since modulation of IL-18 function is likely to be more effective in conditions with a minor role for IFNγ [18].

IL-18 is a known component of the human systemic inflammatory response facilitating T-cell responses and natural killer cell activation [35]. Consequently, high IL-18 levels may also affect post-transplant immune reconstitution. Furthermore, in patients with immune thrombocytopenia, Shan et al. [36,37] demonstrated elevated plasma levels of IL-18 but not IL-18BP during active stages of the disease, proposing also a role for IL-18/IL-18BP imbalance in the pathogenesis and course of immune thrombocytopenia. In our study, we have focused on myeloid reconstitution. Detailed information on post-transplant lymphocyte subset counts was not available. However, it should be noted that pre-conditioning IL-18 levels (i.e., prior to the immune challenge of alloSCT) were also associated with lower pre-conditioning platelet counts. In addition, in the training set, high IL-18 levels measured in the early peri-transplant period (day 0–3), which is characterized by deep aplasia and strong immunosuppressive effects of the starting GVHD-prophylaxis, were also correlated to both delayed platelet and neutrophil recovery. Therefore, although immune effects of IL-18 in the sense of enhanced or abnormal T cell responses triggering immunity against platelets cannot be ruled out, the associations of high IL-18 with post-transplant myeloid reconstitution are more likely to involve impaired hematopoietic stem cell function.

To the best of our knowledge, in vivo effects of IL-18 on hematologic recovery in allografted patients have not been studied so far. In septic patients, serum concentrations of IL-18 were negatively correlated with platelet counts suggesting a role of IL-18 in the development of severe thrombocytopenia during sepsis [38]. Recently, Korpelainen and colleagues [39] in their study on hematological patients receiving intensive chemotherapy found an inverse correlation between IL-18 and leukocyte counts and an association with complicated courses of febrile neutropenia in the subgroup of autologous stem cell recipients with lymphoma. Consequently, it is tempting to speculate that IL-18 neutralization may represent a means to enhance hematopoietic engraftment and/or reduce occurrence of cytopenias in patients with elevated IL-18 levels.

Among the available inhibitors of IL-18 function, Tadekinig alfa, which is a recombinant human IL-18BP, may represent the most promising agent for IL-18 modulation in terms of post-transplant graft recovery. Tadekinig alfa has been investigated in a phase II, open-label study on patients with adult-onset Still’s disease (AOSD) showing a favorable safety profile and early signs of clinical efficacy [40]. In addition, in a recent report, prolonged administration of Tadekinig alfa was safe and shown to be associated with a marked decrease in circulating levels of free IL-18 and improvement of AOSD disease manifestations [41]. The results presented here may provide a rationale for clinical studies exploring the efficacy of Tadekinig alfa in the setting of alloSCT.

Interestingly, platelets themselves may be viewed as versatile intravascular effectors involved in various mechanisms to promote immune responses and are thus increasingly recognized as critical determinants of host defense against infection [42,43]. In septic patients, and similar to IL-18 [14,15], thrombocytopenia has been demonstrated as an independent and complementary risk factor for mortality in multiple studies [43]. In our patients, the persistence of thrombocytopenia ≤20/nL beyond day +28 post-transplant in the absence of relapse was a strong predictor of early non-relapse and overall mortality, adding to the existing evidence supporting the adverse prognostic significance of a delayed platelet recovery [2,5,6,7,8,9]. To this end, linking high IL-18 to delayed platelet recovery in allografted patients, the findings presented here may provide an explanation for our previous observation that increased levels of IL-18 are associated with excess NRM due to fatal infectious complications [16].

Obviously, the findings of this study have to be seen in light of some limitations. First, the retrospective and observational design makes the findings susceptible to bias and unknown confounders. Second, the lack of day 0–3 biomarkers levels for the confirmation cohort needs to be acknowledged. Finally, as with all observational studies, we cannot provide evidence for a causal effect of elevated IL-18 levels on hematopoietic recovery after alloSCT, and therefore the results need to be interpreted with caution.

## 4. Materials and Methods

### 4.1. Patients

Adult patients who were allografted between 2004 and 2015 at the University Hospital Heidelberg, Germany, and had provided permission for sample and data collection comprised the training cohort (*n* = 714). In the training cohort, pre-conditioning serum samples (obtained prior to the start of the conditioning regimen) were available for 602 patients, day 0–3 sera could be retrieved for 306 patients (overlapping patients between pre-conditioning and day 0–3, *n* = 194).

The confirmation cohort consisted of 673 adult patients who underwent alloSCT at the Department of Bone Marrow Transplantation of the University Hospital Essen, Germany, between 2009 and 2013 and had pre-conditioning serum samples available. Written informed consent according to the Declaration of Helsinki was obtained for all patients, and the local ethics committees had approved the sample (ethics committee approval number is 120/2002) and data collection. Patient and outcome data were obtained from medical records and chart review. Disease stage prior to alloSCT and the hematopoietic cell transplantation specific comorbidity index (HCT-CI) was assessed by applying published criteria [19,44]. 

### 4.2. Assessment of Cytokine Serum Levels

Serum samples were collected in gel tubes and cryopreserved at −80 °C. Serum levels of total IL-18, IL-18BP, IFNγ as well as the IFNγ-inducible CXC family chemokines CXCL9 and CXCL10 were assessed by ELISA using commercial kits (DuoSet, R&D Systems, Wiesbaden, Germany) according to the manufacturer’s instructions. The concentration of free IL-18 was calculated as described previously [16]. All values were given in pg/mL.

### 4.3. Definitions and Statistical Analysis

Continuous and categorical variables of patient characteristics were compared using the Mann–Whitney U test and the χ2 test, respectively.

Platelet and absolute neutrophil counts were assessed prior to conditioning and at defined time-points post-transplant (day +14, day +28, day +50, day +100 and year +1). The first (Q1) and fourth quartile (Q4) of the distributions of total IL-18 and free IL-18 were used to stratify patients in low and high IL-18 groups and the median platelet and neutrophil counts were compared with regard to pre-conditioning and day 0–3 levels of total IL-18 and free IL-18. Patients who experienced relapse of the underlying malignancy were excluded from analysis of subsequent landmarks. In addition, correlations between neutrophil and platelet counts were also assessed by employing Spearman’s rank correlation coefficients.

To evaluate associations of cytokines levels with post-transplant hematopoietic recovery, platelet and absolute neutrophil counts (ANC) at defined landmarks were analyzed. For platelets, a delay in recovery was defined as platelets ≤20/nL at day +28, ≤50/nL at day +100 and ≤150/nL at year +1. For neutrophils, a delay in recovery was defined as ANC ≤1/nL at day +28. 

In absence of established reference ranges, cytokine concentrations were analyzed as continuous variables. Since all cytokine serum levels showed a left-skewed distribution, data were log2 transformed. Univariate logistic regression was used to evaluate the association of each cytokine with the outcome of interest. Odds ratios (OR) from these models refer to the increase in odds of the outcome for a twofold increase in the corresponding cytokine. In addition, receiver operating characteristic (ROC) curve analysis with calculation of the area under the curve (AUC) was performed. The results were displayed in tables showing OR and AUC estimates together with the 95% confidence interval (95% CI) and the *p* values.

To adjust for known confounders, multivariable logistic regression was used for the effects of total and free IL-18 including donor HLA-matching, stem cell source, anti-thymocyte globulin (ATG) use, type of conditioning, and disease status at transplant as covariates. Model calibration was evaluated by using the Hosmer-Lemeshow goodness-of-fit test [22].

For the association of delayed platelet recovery on day +28 with alloSCT outcome, overall survival (OS), time to relapse, and NRM (death in the absence of prior relapse) within the first year post-landmark were analyzed from day +28 post-transplant to the appropriate endpoint. NRM and recurrence of the underlying malignancy were considered as competing events and cumulative incidence functions were implemented [45]. For multivariable analysis of predictors of OS, NRM and relapse after day +28, Cox proportional hazard regression models were performed including the covariates specified above and age as an additional confounder [46].

Calculations were done using IBM^®^ SPSS^®^ Statistics, Version 24.0.0 (IBM, Armonk, NY, USA) and the statistical software environment R, version 3.3.2 (R Foundation, Vienna, Austria) together with the R packages ‘survplot’ version 0.0.7, ‘rms’ version 5.1-2, ‘cmprsk’ version 2.2-7, ‘survival’ version 2.42-6. All statistical tests were two-sided. Effects were estimated with 95% confidence interval (95% CI). Results with *p* values < 0.05 were considered to be statistically significant.

## 5. Conclusions

The results of the present study demonstrate an association of high IL-18 serum levels with delayed platelet recovery in patients undergoing alloSCT. Given the functions of IL-18 in regulating HSPC quiescence, the mechanisms underlying this association are likely to involve direct and IFNγ-independent inhibitory effects of IL-18 on hematopoietic progenitor proliferation in allografted patients. The findings may provide a rationale to explore modulation of IL-18 for improving outcomes of alloSCT. 

## Figures and Tables

**Table 1:** Baseline patient characteristics and cytokine serum levels.

Parameter	Training Cohort (*n* = 714)	Confirmation Cohort (*n* = 673)	*p*
Age (years) at alloSCT (median, IQR)	54 (45–61)	54 (44–61)	0.71
Patient sex, *n* (%) Female Male	274 (38) 440 (62)	314 (47) 359 (53)	0.002
Disease stage before alloSCT^a^, *n* (%) Early Intermediate Late	251 (35) 204 (29) 259 (36)	304 (45) 235 (35) 134 (20)	<0.001
Diagnosis, *n* (%) AML MDS/MPN Lymphoma ALL MM	261 (37) 121 (17) 222 (31) 28 (4) 82 (11)	344 (51) 134 (20) 99 (15) 70 (10) 26 (4)	<0.001
Conditioning^b^, *n* (%) RIC MAC	649 (91) 65 (9)	531 (79) 142 (21)	<0.001
Donor, *n* (%) RD MUD MMUD	207 (29) 359 (50) 148 (21)	192 (29) 319 (47) 162 (24)	0.31
Donor sex, *n* (%) Female Male	229 (32) 485 (68)	232 (34) 441 (66)	0.34
ATG treatment, *n* (%) No Yes	222 (31) 492 (69)	284 (42) 389 (58)	<0.001
GVHD prophylaxis, *n* (%) CNI + MTX CNI + MMF	216 (30) 498 (70)	632 (94) 41 (6)	<0.001
Stem cell source, *n* (%) Peripheral blood Bone marrow	673 (94) 41 (6)	603 (90) 70 (10)	0.001
Pre-conditioning total IL-18 (pg/mL) (median, IQR)	627 (437–930)	692 (462–1026)	0.04
Pre-conditioning IL-18BP (pg/mL) (median, IQR)	5200 (4313–5987)	11,404 (9282–12,101)	<0.001
Pre-conditioning free IL-18 (pg/mL) (median, IQR)	414 (266–658)	414 (283–608)	0.96
Pre-conditioning CXCL9 (pg/mL) (median, IQR)	199 (85–679)	211 (114–382)	0.92
Pre-conditioning CXCL10 (pg/mL) (median, IQR)	85 (45–186)	–	–
Pre-conditioning IFNγ (pg/mL) (median, IQR)	7.5 (2.0–18.7)	–	–

Abbreviations: ALL, acute lymphoblastic leukemia; alloSCT, allogeneic stem cell transplantation; AML, acute myeloid leukemia; ATG, anti-thymocyte globulin; CNI, calcineurin inhibitor; CXCL, chemokine (C-X-C motif) ligand; IFNγ, interferon gamma; IL-18, interleukin-18; IL-18BP, interleukin-18 binding protein; IQR, interquartile range; HLA, human leukocyte antigen; MAC, myeloablative conditioning; MDS, myelodysplastic syndrome; MM, multiple myeloma; MMF, mycophenolate mofetil; MMUD, mismatched unrelated donor; MPN, myeloproliferative neoplasm; MTX, methotrexate; MUD, matched unrelated donor; RD, related donor; RIC, reduced intensity conditioning. ^a^ According to Gratwohl et al. [19]. ^b^ According to Bacigalupo et al. [20] and Bornhäuser et al. [21].

**Table 2:** Associations of pre-conditioning cytokine serum levels with platelet and neutrophil recovery and corresponding predictive values (training cohort).

	Odds Ratio (OR) and Area under the ROC Curve (AUC) per log2 Increase* in Pre-Conditioning Cytokine			
Cytokine	Platelets Day +28 ≤20/nL (*n* = 41) vs. >20/nL (*n* = 512)	Platelets Day +50 ≤100/nL (*n* = 117) vs. >100/nL (*n* = 391)	Platelets Year +1 ≤150/nL (*n* = 84) vs. >150/nL (*n* = 274)	ANC Day +28 ≤1/nL (*n* = 43) vs. >1/nL (*n* = 331)
Total IL-18 OR (95% CI), *p* AUC (95% CI), *p*	1.56 (1.20–2.03), 0.001 0.68 (0.60–0.76), <0.001	1.52 (1.24–1.86), <0.001 0.63 (0.58–0.69), <0.001	1.28 (1.01–1.61), 0.04 0.57 (0.50-0.64), 0.04	1.61 (1.23–2.13), 0.001 0.65 (0.56–0.74), 0.002
IL-18BP OR (95% CI), *p* AUC (95% CI), *p*	0.78 (0.60–1.02), 0.07 0.46 (0.38–0.54), 0.41	0.98 (0.86–1.11), 0.72 0.50 (0.45–0.56), 0.94	0.96 (0.82–1.10), 0.55 0.51 (0.44–0.58), 0.75	0.86 (0.68–1.09), 0.20 0.50 (0.41–0.58), 0.91
Free IL-18 OR (95% CI), *p* AUC (95% CI), *p*	1.72 (1.32–2.23), <0.001 0.72 (0.65–0.79), <0.001	1.45 (1.20–1.75), <0.001 0.64 (0.58–0.69), <0.001	1.23 (1.01–1.50), 0.04 0.56 (0.49–0.63), 0.12	1.81 (1.36–2.41), <0.001 0.67 (0.58–0.76), <0.001
CXCL9 OR (95% CI), *p* AUC (95% CI), *p*	0.93 (0.81–1.06), 0.27 0.43 (0.34–0.52), 0.13	0.99 (0.91–1.09), 0.91 0.50 (0.44–0.56), 0.96	0.98 (0.88–1.08), 0.62 0.47 (0.41–0.54), 0.42	1.02 (0.88–1.17), 0.80 0.50 (0.42–0.58), 0.98
CXCL10 OR (95% CI), *p* AUC (95% CI), *p*	1.01 (0.87–1.18), 0.90 0.50 (0.42–0.59), 0.93	0.98 (0.88–1.08), 0.63 0.47 (0.41–0.53), 0.39	0.95 (0.85–1.06), 0.38 0.47 (0.40–0.53), 0.36	1.02 (0.87–1.18), 0.83 0.50 (0.41–0.59), 0.96
IFNγ OR (95% CI), *p* AUC (95% CI), *p*	1.06 (0.91–1.25), 0.44 0.54 (0.44–0.65), 0.39	1.01 (0.91–1.12), 0.86 0.50 (0.44–0.57), 0.94	0.90 (0.79–1.01), 0.08 0.43 (0.36–0.50), 0.05	1.07 (0.91–1.27), 0.39 0.48 (0.39–0.57), 0.55

*Each one unit increase in log2 corresponds to a doubling in the corresponding cytokine level. Abbreviations: ANC, absolute neutrophil count; AUC, area under the ROC curve; CI, confidence interval; CXCL, chemokine (C-X-C motif) ligand; IFNγ, interferon gamma; IL-18, interleukin-18; IL-18BP, interleukin-18 binding protein; OR, odds ratio; ROC, receiver operating characteristic.

**Table 3:** Associations of day 0–3 cytokine serum levels with platelet and neutrophil recovery and corresponding predictive values (training cohort).

	Odds Ratio (OR) and Area under the ROC Curve (AUC) per log2 Increase* in Day 0–3 Cytokine			
Cytokine	Platelets Day +28 ≤20/nL (*n* = 28) vs. >20/nL (*n* = 257)	Platelets Day +50 ≤100/nL (*n* = 67) vs. >100/nL (*n* = 196)	Platelets Year +1 ≤150/nL (*n* = 55) vs. >150/nL (*n* = 125)	ANC Day +28 ≤1/nL (*n* = 31) vs. >1/nL (*n* = 175)
Total IL-18 OR (95% CI), *p* AUC (95% CI), *p*	2.27 (1.52–3.39), <0.001 0.70 (0.59–0.81), 0.001	1.95 (1.40–2.72), <0.001 0.68 (0.61–0.75), <0.001	1.72 (1.19–2.48), 0.004 0.63 (0.55–0.72), 0.005	2.07 (1.34–3.21), 0.001 0.67 (0.56–0.79), 0.002
IL-18BP OR (95% CI), *p* AUC (95% CI), *p*	1.50 (0.76–2.96), 0.29 0.55 (0.45-0.66), 0.35	1.70 (1.02–2.84), 0.04 0.59 (0.52–0.66), 0.03	1.03 (0.62–1.68), 0.92 0.49 (0.40–0.58), 0.82	1.45 (0.75–2.78), 0.27 0.58 (0.48–0.68), 0.15
Free IL-18 OR (95% CI), *p* AUC (95% CI), *p*	2.12 (1.45–3.10), <0.001 0.70 (0.58–0.81), 0.001	1.86 (1.34–2.59), <0.001 0.68 (0.61–0.75), <0.001	1.74 (1.21–2.48), 0.003 0.64 (0.56–0.73), 0.002	1.95 (1.28–2.96), 0.002 0.66 (0.55–0.78), 0.004
CXCL9 OR (95% CI), *p* AUC (95% CI), *p*	1.21 (1.01–1.46), 0.04 0.61 (0.49–0.72), 0.06	1.09 (0.97–1.21), 0.14 0.56 (0.47–0.64), 0.18	0.98 (0.86–1.12), 0.77 0.47 (0.38–0.57), 0.56	1.05 (0.89–1.23), 0.58 0.54 (0.42–0.66), 0.49
IFNγ OR (95% CI), *p* AUC (95% CI), *p*	1.20 (0.99–1.44), 0.06 0.61 (0.50–0.71), 0.07	1.29 (1.11–1.51), 0.001 0.66 (0.58–0.74), <0.001	1.01 (0.85–1.19), 0.93 0.52 (0.42–0.62), 0.72	1.20 (0.99–1.46), 0.06 0.64 (0.53–0.75), 0.02

*Each one unit increase in log2 corresponds to a doubling in the corresponding cytokine level. Abbreviations: ANC, absolute neutrophil count; AUC, area under the ROC curve; CI, confidence interval; CXCL, chemokine (C-X-C motif) ligand; IFNγ, interferon gamma; IL-18, interleukin-18; IL-18BP, interleukin-18 binding protein; OR, odds ratio; ROC, receiver operating characteristic.

**Table 4:** Multivariable logistic regression analysis of pre-conditioning total and free IL-18 serum levels with regard to platelet and neutrophil recovery (training cohort).

Covariate, Effect	Platelets Day +28 ≤20/nL (*n* = 41) vs. >20/nL (*n* = 512)		Platelets Day +50 ≤100/nL (*n* = 117) vs. >100/nL (*n* = 391)		Platelets Year +1 ≤150/nL (*n* = 84) vs. >150/nL (*n* = 274)		ANC Day +28 ≤1/nL (*n* = 43) vs. >1/nL (*n* = 331)	
	**Model with total IL-18,** **aOR (95% CI), *p***	**Model with ** **free IL-18,** **aOR (95% CI), *p***	**Model with total IL-18,** **aOR (95% CI), *p***	**Model with ** **free IL-18,** **aOR (95% CI), *p***	**Model with total IL-18,** **aOR (95% CI), *p***	**Model with ** **free IL-18,** **aOR (95% CI), *p***	**Model with total IL-18,** **aOR (95% CI), *p***	**Model with** **free IL-18,** **aOR (95% CI), *p***
Total IL-18, per log2 increase *	1.61 (1.21–2.15), 0.001	–	1.52 (1.22–1.88), <0.001	–	1.28 (1.01–1.63), 0.04	–	1.63 (1.20–2.21), 0.002	–
Free IL-18, per log2 increase *	–	1.78 (1.34–2.36), <0.001	–	1.43 (1.18–1.74), 0.002	–	1.22 (0.99–1.50), 0.06	–	1.76 (1.30–2.39), <0.001
Donor, mismatched vs. matched	3.43 (1.70–6.92), 0.001	3.45 (1.72–7.04), 0.001	2.69 (1.66–4.37), <0.001	2.68 (1.65–4.35), <0.001	1.21 (0.64–2.29), 0.56	1.22 (0.65–2.30), 0.54	1.84 (0.86–3.93), 0.11	1.89 (0.88–4.05), 0.10
Stem cell source, PB vs. BM	0.34 (0.12–0.98), 0.05	0.32 (0.11–0.94), 0.04	0.74 (0.30–1.86), 0.53	0.75 (0.30–1.88), 0.54	0.19 (0.07–0.54), 0.002	0.19 (0.07–0.54), 0.002	0.13 (0.04–0.41), 0.001	0.13 (0.04–0.42), 0.001
ATG, yes vs. no	1.42 (0.59–3.41), 0.43	1.45 (0.60–3.52), 0.41	1.64 (0.95–2.84), 0.08	1.72 (1.00–2.97), 0.05	0.96 (0.54–1.70), 0.88	0.97 (0.55–1.73), 0.93	4.15 (1.45–11.89), 0.008	4.12 (1.42–11.92), 0.009
Conditioning, MAC vs. RIC	1.50 (0.53–4.23), 0.45	1.43 (0.51–4.07), 0.50	1.36 (0.64–2.91), 0.42	1.27 (0.60–2.70), 0.54	1.33 (0.55–3.24), 0.53	1.27 (0.52–3.08), 0.60	2.78 (1.00–7.75), 0.05	2.86 (1.01–8.07), 0.05
Disease stage, high vs intermediate/low	0.73 (0.36–1.47), 0.38	0.69 (0.34–1.40), 0.30	1.38 (0.88–2.15), 0.16	1.34 (0.86–2.09), 0.20	1.52 (0.89–2.60), 0.12	1.50 (0.88–2.55), 0.14	1.51 (0.76–3.03), 0.24	1.44 (0.72–2.91), 0.30
*Goodness-of- fit test* ^†^	*Χ^2^ = 6.43 (8 df),* *p = 0.60*	*Χ^2^ = 6.83 (8 df),* *p = 0.56*	*Χ^2^ = 10.10 (8 df),* *p = 0.29*	*Χ^2^ = 11.26 (8 df),* *p = 0.19*	*Χ^2^ = 6.16 (8 df),* *p = 0.63*	*Χ^2^ = 8.24 (8 df),* *p = 0.41*	*Χ^2^ = 7.47 (8 df),* *p = 0.49*	*Χ^2^ = 10.08 (8 df),* *p = 0.26*

Abbreviations: ANC, absolute neutrophil count; aOR, adjusted odds ratio; ATG, antithymocyte globulin; CI, confidence interval; df, degrees of freedom; IL-18, interleukin-18; MAC, myeloablative conditioning; RIC, reduced intensity conditioning. * Each one unit increase in log2 corresponds to a doubling in the corresponding cytokine level. ^†^ Hosmer-Lemeshow test [22].

**Table 5:** Associations of pre-conditioning cytokine serum levels with platelet and neutrophil recovery and corresponding predictive values (confirmation cohort).

Cytokine	Odds Ratio (OR) and Area under the ROC Curve (AUC) per log2 Increase* in Pre-Conditioning Cytokine	
	Platelets Day +28 ≤20/nL (*n* = 100) vs. >20/nL (*n* = 561)	ANC Day +28 ≤1/nL (*n* = 161) vs. >1/nL (*n* = 500)
Total IL-18 OR (95% CI), *p* AUC (95% CI), *p*	1.42 (1.07–1.90), 0.02 0.58 (0.52–0.65), 0.008	1.15 (0.91–1.45), 0.26 0.54 (0.49–0.59), 0.13
IL-18BP OR (95% CI), *p* AUC (95% CI), *p*	2.60 (1.45–4.66), 0.001 0.60 (0.54–0.67), 0.001	1.20 (0.79–1.82), 0.39 0.56 (0.51–0.61), 0.02
Free IL-18 OR (95% CI), *p* AUC (95% CI), *p*	1.31 (0.98–1.76), 0.07 0.56 (0.50–0.63), 0.04	1.10 (0.86–1.40), 0.46 0.53 (0.48–0.58), 0.28
CXCL9 OR (95% CI), *p* AUC (95% CI), *p*	0.90 (0.71–1.14), 0.38 0.45 (0.35–0.56), 0.34	0.97 (0.81–1.16), 0.70 0.48 (0.40–0.55), 0.53

* Each one unit increase in log2 corresponds to a doubling in the corresponding cytokine level. Abbreviations: ANC, absolute neutrophil count; AUC, area under the ROC curve; CI, confidence interval; CXCL, chemokine (C-X-C motif) ligand; IFNγ, interferon gamma; IL-18, interleukin-18; IL-18BP, interleukin-18 binding protein; OR, odds ratio; ROC, receiver operating characteristic.

**Table 6:** Multivariable logistic regression analysis of pre-conditioning total and free IL-18 serum levels with regard to platelet and neutrophil recovery (confirmation cohort).

Covariate, Effect	Platelets Day +28 ≤20/nL (*n* = 100) Vs. >20/nL (*n* = 561)		ANC Day +28 ≤1/nL (*n* = 161) Vs. >1/nL (*n* = 500)	
	Model with total IL-18, aOR (95% CI), *p*	Model with free IL-18, aOR (95% CI), *p*	Model with total IL-18, aOR (95% CI), *p*	Model with free IL-18, aOR (95% CI), *p*
Total IL-18, per log2 increase *	1.80 (1.29–2.50), <0.001	–	1.28 (0.99–1.65), 0.06	–
Free IL-18, per log2 increase *	–	1.66 (1.19–2.31), 0.003	–	1.22 (0.94–1.59), 0.14
Donor, mismatched vs matched	1.56 (0.94–2.57), 0.08	1.56 (0.95–2.58), 0.08	0.84 (0.54–1.31), 0.44	0.84 (0.54–1.32), 0.45
Stem cell source, PB vs BM	0.51 (0.24–1.10), 0.09	0.52 (0.25–1.11), 0.09	0.19 (0.11–0.35), <0.001	0.20 (0.11–0.35), <0.001
ATG, yes vs. no	3.59 (2.04–6.32), <0.001	3.60 (2.05–6.33), <0.001	2.96 (1.91–4.60), <0.001	2.96 (1.90–4.59), <0.001
Conditioning, MAC vs. RIC	2.17 (1.26–3.76), 0.006	2.18 (1.26–3.76), 0.005	1.35 (0.85–2.16), 0.20	1.35 (0.85–2.15), 0.21
Disease stage, high vs. intermediate/low	2.92 (1.70–5.04), <0.001	2.98 (1.73–5.13), <0.001	2.06 (1.29–3.29), 0.002	2.08 (1.30–3.32), 0.002
*Goodness-of- fit test* ^†^	*Χ^2^ = 4.84 (8 df), p = 0.78*	*Χ^2^ = 5.31 (8 df), p = 0.72*	*Χ^2^ = 7.91 (8 df), p = 0.44*	*Χ^2^ = 12.35 (8 df), p = 0.14*

Abbreviations: ANC, absolute neutrophil count; aOR, adjusted odds ratio; ATG, antithymocyte globulin; CI, confidence interval; df, degrees of freedom; IL-18, interleukin-18; MAC, myeloablative conditioning; RIC, reduced intensity conditioning. * Each one unit increase in log2 corresponds to a doubling in the corresponding cytokine level. ^†^ Hosmer-Lemeshow test [22].

## Supplementary Materials

The following are available online at https://www.mdpi.com/2072-6694/12/10/2789/s1. Figure S1: Associations of pre-conditioning total IL-18 serum levels with pre-transplant patient characteristics and pre-conditioning CRP and ferritin levels, Figure S2: Associations of pre-conditioning free IL-18 serum levels with pre-transplant patient characteristics and pre-conditioning CRP and ferritin levels, Figure S3: Associations of day 0–3 total IL-18 serum levels with pre-transplant patient characteristics and pre-conditioning CRP and ferritin levels, Figure S4: Associations of day 0–3 free IL-18 serum levels with pre-transplant patient characteristics and pre-conditioning CRP and ferritin levels, Table S1: Baseline patient characteristics of the day 0–3 cohort, Table S2: Pre-conditioning versus day 0–3 cytokine levels (median, IQR) in the training cohort: (A) all patients, (B) overlap cohort (*n* = 194), Table S3: Correlation (Spearman’s rank correlation coefficients) of pre-conditioning and day 0–3 total and free IL-18 serum levels with platelet and neutrophil counts pre-conditioning and at different time-points following alloSCT: training cohort (A), confirmation cohort (B), Table S4: Multivariable logistic regression analysis of day 0–3 total and day 0–3 free IL-18 serum levels with regard to platelet and neutrophil recovery (training cohort), Table S5: Multivariable analysis of day +28 platelets and predictors of overall survival (OS), non-relapse mortality (NRM) and relapse within the first year following day +28 after allogeneic stem cell transplantation (complete case analysis).
