# Supplementary material for: Interleukin-18 and Hematopoietic Recovery after Allogeneic Stem Cell Transplantation
Source: Cancers (Basel). 2020 Sep 28;12(10):2789. doi: 10.3390/cancers12102789 (PMC7601738; doi:10.3390/cancers12102789)
Supplement: Supplementary file 1 [file cancers-12-02789-s001.pdf]

cancers

Supplementary Materials: Interleukin-18 and Hematopoietic Recovery after Allogeneic Stem Cell Transplantation

Aleksandar Radujkovic, Lambros Kordelas, Rashit Bogdanov, Carsten Müller-Tidow, Dietrich W. Beelen, Peter Dreger and Thomas Luft

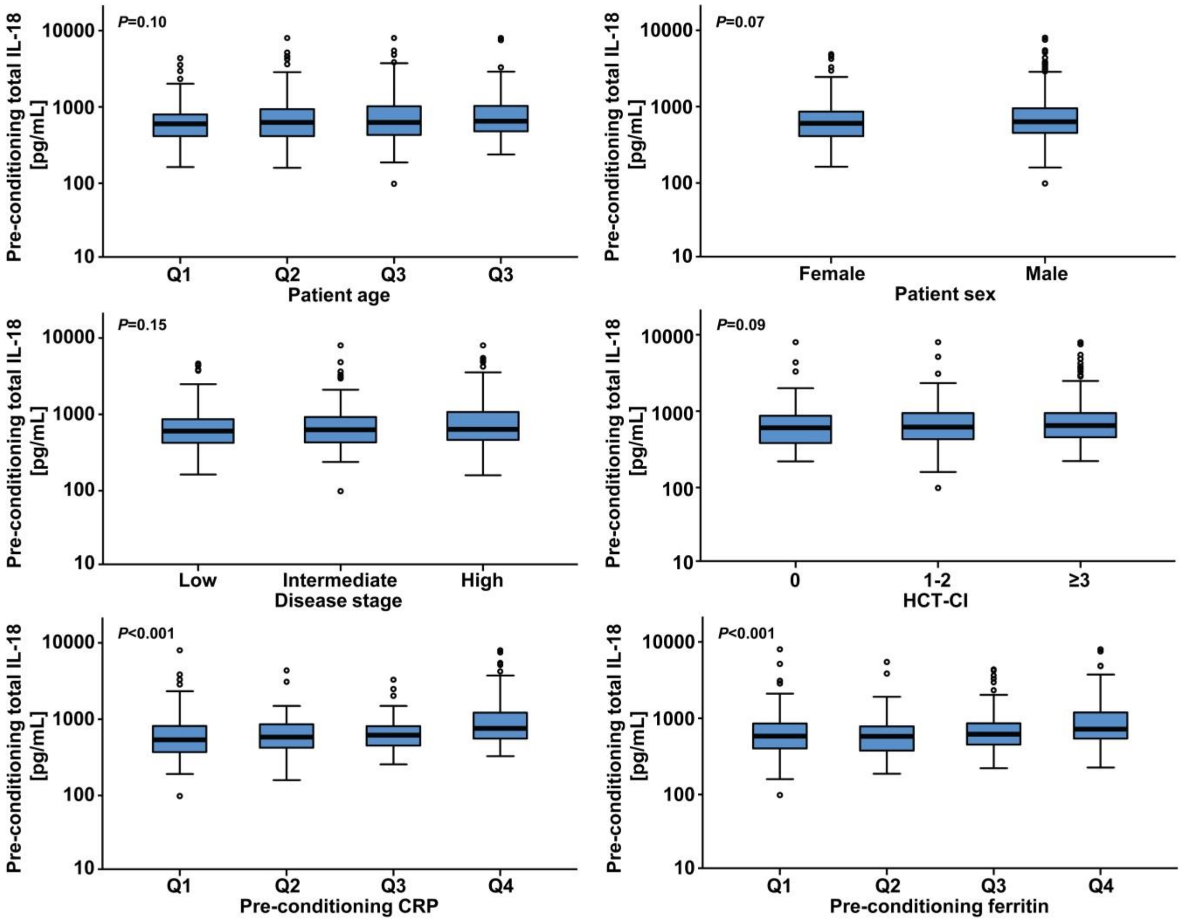

Figure S1. Associations of pre-conditioning total IL-18 serum levels with pre-transplant patient characteristics and pre-conditioning CRP and ferritin levels.

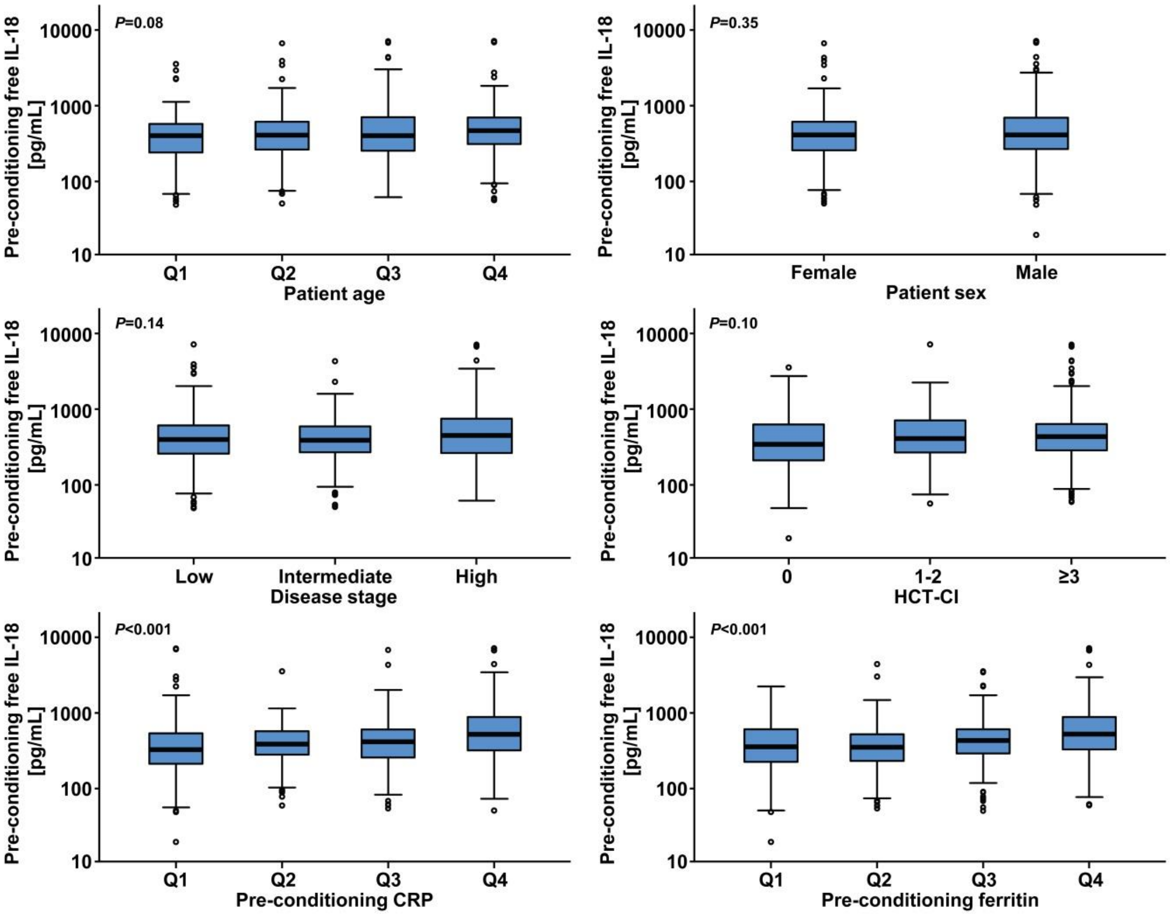

Figure S2. Associations of pre-conditioning free IL-18 serum levels with pre-transplant patient characteristics and pre-conditioning CRP and ferritin levels.

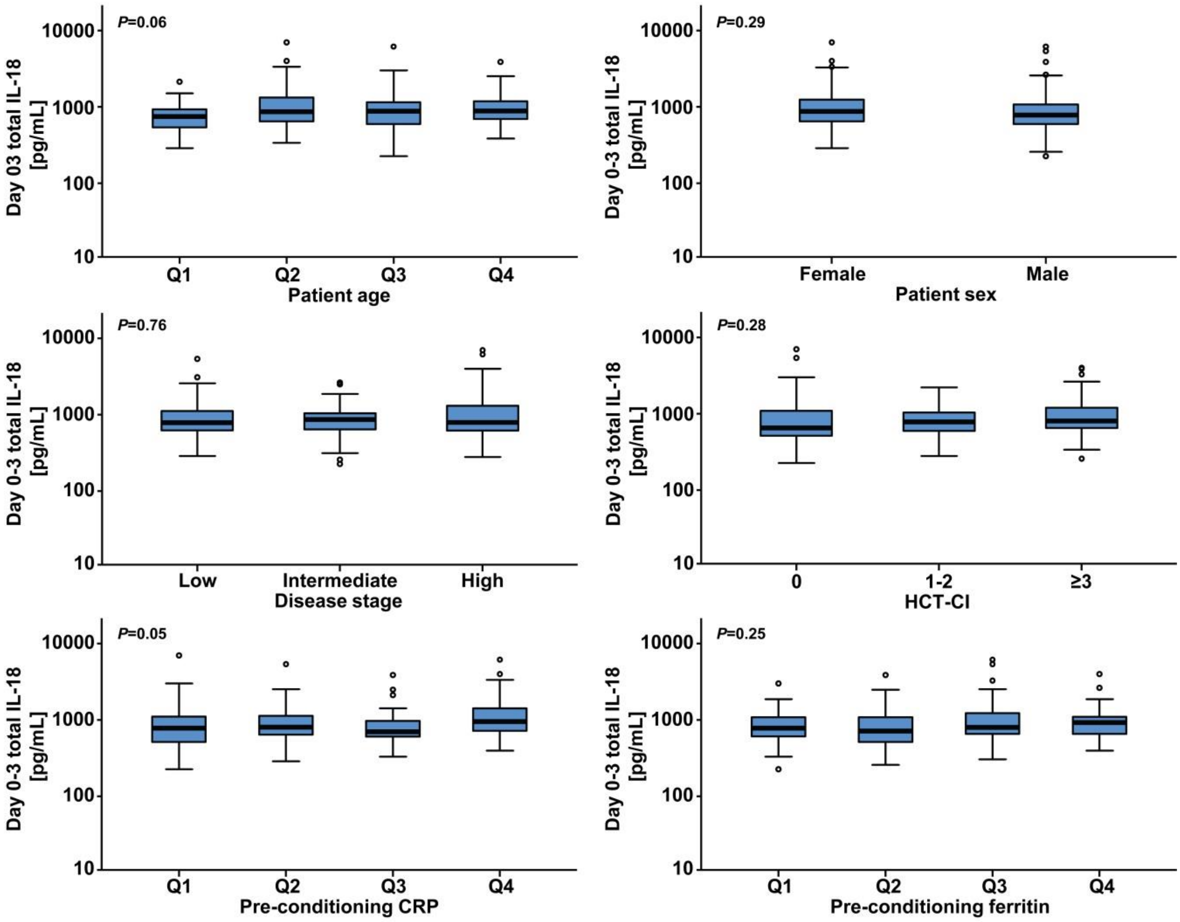

Figure S3. Associations of day 0–3 total IL-18 serum levels with pre-transplant patient characteristics and pre-conditioning CRP and ferritin levels.

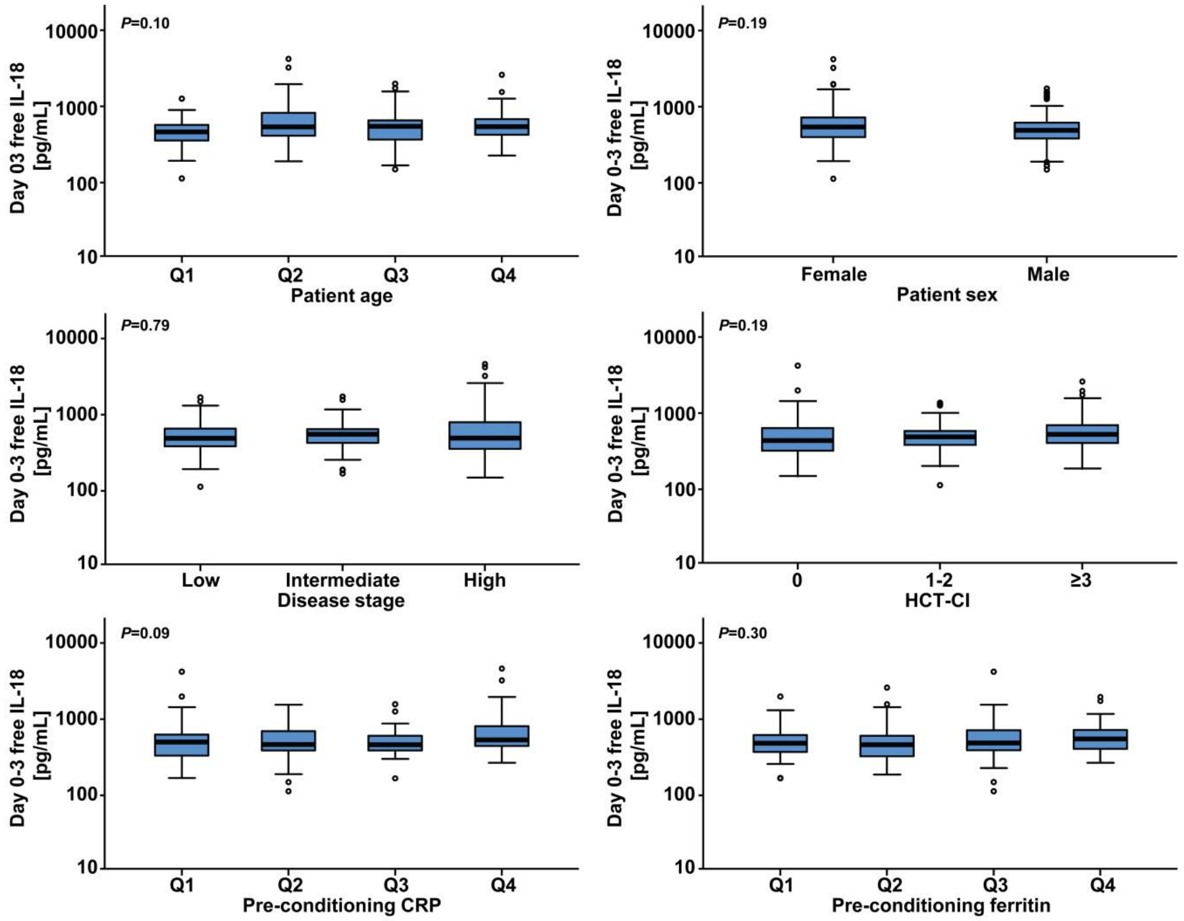

Figure S4. Associations of day 0–3 free IL-18 serum levels with pre-transplant patient characteristics and pre-conditioning CRP and ferritin levels.

Table S1. Baseline patient characteristics of the day 0–3 cohort.

Parameter	Entire Cohort (*n* = 714)	Day 0–3 Cohort (*n* = 306)	*p*
Age [years] at alloSCT (median, IQR)	54 (45-61)	54 (44-61)	0.77
Patient sex, *n* (%)			
Female	274 (38)	113 (37)	0.66
Male	440 (62)	193 (66)	
Disease stage before alloSCT, *n* (%)			
Early	251 (35)	109 (36)	0.84
Intermediate	204 (29)	82 (27)	
Late	259 (36)	115 (38)	
Diagnosis, *n* (%)			
AML	261 (37)	114 (37)	0.90
MDS/MPN	121 (17)	45 (15)	
Lymphoma	222 (31)	95 (31)	
ALL	28 (4)	14 (5)	
MM	82 (11)	38 (12)	
Conditioning, *n* (%)			0.19
RIC	649 (91)	270 (88)	
MAC	65 (9)	36 (12)	
Donor, *n* (%)			
RD	207 (29)	84 (27)	0.82
MUD	359 (50)	154 (50)	
MMUD	148 (21)	68 (22)	
Donor sex, *n* (%)			
Female	229 (32)	98 (32)	0.99
Male	485 (68)	208 (68)	
ATG treatment, *n* (%)			
No	222 (31)	89 (29)	0.52
Yes	492 (69)	217 (71)	
GVHD prophylaxis, *n* (%)			
CNI + MTX	216 (30)	98 (32)	0.57
CNI + MMF	498 (70)	208 (68)	
Stem cell source, *n* (%)			
Peripheral blood	673 (94)	291 (95)	0.59
Bone marrow	41 (6)	15 (5)	

Abbreviations: ALL, acute lymphoblastic leukemia; alloSCT, allogeneic stem cell transplantation; AML, acute myeloid leukemia; ATG, anti-thymocyte globulin; CNI, calcineurin inhibitor; IQR, interquartile range; HLA, human leukocyte antigen; MAC, myeloablative conditioning; MDS, myelodysplastic syndrome; MM, multiple myeloma; MMF, mycophenolate mofetil; MMUD, mismatched unrelated donor; MPN, myeloproliferative neoplasm; MTX, methotrexate; MUD, matched unrelated donor; RD, related donor; RIC, reduced intensity conditioning.

Table S2. Pre-conditioning versus day 0–3 cytokine levels (median, IQR) in the training cohort: (**A**) all patients, (**B**) overlap cohort (*n* = 194).

**A**				
**Cytokine**	**Pre-conditioning (*n* = 602)**	**Day 0–3 (*n* = 306)**	***p***	
Total IL-18	627 (437-930)	797 (598-1173)	<0.001	
IL-18BP	5200 (4313-5987)	10438 (8164-12898)	<0.001	
Free IL-18	414 (266-658)	489 (382-699)	<0.001	
CXCL9	199 (85-679)	195 (67-505)	0.05	
IFNγ	7.5 (2.0-18.7)	7.7 (3.0-16.6)	0.45	
**B**				
**Cytokine**	**Pre-conditioning (*n* = 194)**	**Day 0–3 (*n* = 194)**	***p***	**Rho*, *p***
Total IL-18	626 (442–874)	799 (621–1173)	<0.001	0.598, <0.001
IL-18BP	5280 (4422–5883)	10338 (8185–12763)	<0.001	0.237, 0.001
Free IL-18	427 (275–644)	504 (389–693)	<0.001	0.493, <0.001
CXCL9	173 (69–473)	181 (49–447)	0.37	0.294, <0.001
IFNγ	8.1 (2.5–17.4)	8.4 (3.1–17.0)	0.85	0.122, 0.10

*Spearman's rho correlation between pre-conditioning and day 0–3 cytokine levels. All values are given in pg/mL. Abbreviations: CXCL, chemokine (C-X-C motif) ligand; IFNγ, interferon gamma; IL-18, interleukin-18; IL-18BP, interleukin-18 binidng protein; IQR, interquartile range.

Table S3. Correlation (Spearman's rank correlation coefficients) of pre-conditioning and day 0-3 total and free IL-18 serum levels with platelet and neutrophil counts pre-conditioning and at different time-points following alloSCT: training cohort (**A**), confirmation cohort (**B**).

**A**								
	**Pre-Conditioning IL-18**				**Day 0–3 IL-18**			
	**Total**		**Free**		**Total**		**Free**	
	**Rho***	***p***	**Rho***	***p***	**Rho***	***p***	**Rho***	***p***
**Platelets**								
Pre	−0.250	<0.001	−0.237	<0.001	-	-	-	-
D+14	−0.115	0.005	−0.085	0.04	−0.246	<0.001	−0.192	0.001
D+28	−0.236	<0.001	−0.221	<0.001	−0.294	<0.001	−0.262	<0.001
D+50	−0.257	<0.001	−0.228	<0.001	−0.296	<0.001	−0.289	<0.001
D+100	−0.193	<0.001	−0.167	<0.001	−0.248	<0.001	−0.231	<0.001
Year+1	−0.165	0.002	−0.161	0.002	−0.211	0.004	−0.219	0.003
**ANC**								
Pre	−0.055	0.35	−0.060	0.30	-	-	-	-
D+14	−0.083	0.06	−0.064	0.16	−0.112	0.07	−0.061	0.32
D+28	−0.018	0.73	−0.013	0.81	−0.227	0.002	−0.224	0.002
D+50	−0.095	0.05	−0.067	0.17	−0.120	0.07	−0.159	0.02
D+100	−0.127	0.008	−0.098	0.04	−0.097	0.15	−0.130	0.05
Year+1	−0.010	0.85	−0.036	0.50	−0.074	0.34	−0.073	0.34

**B**				
	**Pre-Conditioning IL-18**			
	**Total**		**Free**	
	Rho	*p*	Rho	*p*
**Platelets**				
Pre	−0.186	<0.001	−0.183	<0.001
D+28	−0.175	<0.001	−0.143	<0.001
**ANC**				
Pre	−0.071	0.06	−0.089	0.02
D+28	−0.079	0.04	−0.061	0.12

*Spearman rank-correlation coefficients. Abbreviations: ANC, absolute neutrophil count; IL-18, interleukin-18.

Table S4. Multivariable logistic regression analysis of day 0-3 total and day 0-3 free IL-18 serum levels with regard to platelet and neutrophil recovery (training cohort).

**Covariate, Effect**	**Model with Total IL-18, aOR (95% CI), *p***	**Model with Free IL-18, aOR (95% CI), *p***	**Model with Total IL-18, aOR (95% CI), *p***	**Model with Free IL-18, aOR (95% CI), *p***	**Model with Total IL-18, aOR (95% CI), *p***	**Model with Free IL-18, aOR (95% CI), *p***	**Model with Total IL-18, aOR (95% CI), *p***	**Model with Free IL-18, aOR (95% CI), *p***
Total IL-18, per log2 increase*	2.00 (1.24–3.21), 0.004	–	1.80 (1.25–2.57), 0.001	–	1.69 (1.15–2.50), 0.008	–	1.85 (1.13–3.02), 0.02	–
Free IL-18, per log2 increase*	–	1.84 (1.18–2.89), 0.008	–	1.72 (1.21–2.43), 0.002	–	1.73 (1.19–2.51), 0.004	–	1.77 (1.11–2.80), 0.02
Donor, mismatched vs matched	4.10 (1.70–9.88), 0.002	3.93 (1.63–9.44), 0.002	2.31 (1.20–4.47), 0.01	2.29 (1.15–4.30), 0.02	1.36 (0.62–2.97), 0.44	1.29 (0.59–2.84), 0.52	1.62 (0.63–4.15), 0.32	1.59 (0.62–4.07), 0.33
Stem cell source, PB vs BM	0.19 (0.05–0.76), 0.02	0.19 (0.05–0.76), 0.02	0.56 (0.15–2.08), 0.39	0.57 (0.15–2.10), 0.396	0.38 (0.09–1.60), 0.19	0.38 (0.09–1.60), 0.19	0.11 (0.02–0.65), 0.01	0.11 (0.02–0.63), 0.01
ATG, yes vs no	1.98 (0.51–7.64), 0.32	2.26 (0.59–8.63), 0.23	1.69 (0.78–3.65), 0.18	1.90 (0.89–4.05), 0.10	1.16 (0.51–2.64), 0.73	1.27 (0.56–2.86), 0.57	2.21 (0.67–7.27), 0.19	2.54 (0.79–8.19), 0.12
Conditioning, MAC vs RIC	0.30 (0.04–2.49), 0.27	0.29 (0.04–2.39), 0.25	1.07 (0.43–2.67), 0.88	1.04 (0.42–2.58), 0.93	1.39 (0.50–3.84), 0.53	1.42 (0.51–3.93), 0.51	1.32 (0.34–5.15), 0.69	1.29 (0.33–4.99), 0.71
Disease stage, high vs intermediate/low	0.87 (0.34–2.22), 0.77	0.81 (0.35–2.26), 0.81	1.24 (0.66–2.33), 0.50	1.25 (0.67–2.34), 0.48	0.71 (0.32–1.56), 0.40	0.70 (0.32–1.55), 0.38	1.08 (0.46–2.56), 0.86	1.09 (0.46–2.58), 0.84
Goodness-of- fit test	X^2 = *6.33 (8 df), p = 0.61*	X^2 = *6.55 (8 df), p = 0.59*	X^2 = *5.85 (8 df), p = 0.66*	X^2 = *7.53 (8 df), p = 0.48*	X^2 = *15.11 (8 df), p = 0.06*	X^2 = *4.79 (8 df), p = 0.78*	X^2 = *9.47 (8 df), p = 0.30*	X^2 = *7.06 (8 df), p = 0.53*

Abbreviations: ANC, absolute neutrophil count; aOR, adjusted odds ratio; ATG, anti-thymocyte globulin; CI, confidence interval; df, degrees of freedom; IL-18, interleukin-18; MAC, myeloablative conditioning; RIC, reduced intensity conditioning. *Each one unit increase in log2 corresponds to a doubling in the corresponding cytokine level.

Table S5. Multivariable analysis of day +28 platelets and predictors of overall survival (OS), non-relapse mortality (NRM) and relapse within the first year following day +28 after allogeneic stem cell transplantation (complete case analysis).

Covariate	Training Cohort (n = 653) *						Confirmation Cohort (n = 652) **					
	OS		Relapse		NRM		OS		Relapse		NRM	
	HR 95% CI	*p*	HR 95% CI	*p*	HR 95% CI	*p*	HR 95% CI	*p*	HR 95% CI	*p*	HR 95% CI	*p*
Day +28 platelets												
>20/nL	Ref		Ref		Ref		Ref		Ref		Ref	
≤20/nL	1.84 (1.13–3.01)	0.01	1.31 (0.71-2.40)	0.38	2.02 (1.00-4.12)	0.05	2.33 (1.70–3.19)	<0.001	0.74 (0.44–1.21)	0.25	3.25 (2.24–4.72)	<0.001
Disease stage												
Low/intermediate	Ref		Ref		Ref		Ref		Ref		Ref	
High	1.78 (1.31–2.42)	<0.001	1.94 (1.40–2.67)	<0.001	1.29 (0.81–2.05)	0.29	1.35 (1.01–1.82)	0.05	1.65 (1.13–2.41)	0.01	0.98 (0.65–1.47)	0.92
Donor-recipient HLA matching												
Matched	Ref		Ref		Ref		Ref		Ref		Ref	
Mismatched	1.56 (1.10–2.23)	0.01	1.07 (0.71–1.61)	0.76	1.64 (0.96–2.80)	0.07	1.40 (1.06–1.85)	0.02	1.10 (0.75–1.61)	0.64	1.49 (1.06–2.08)	0.02
Stem cell source												
Bone marrow	Ref		Ref		Ref		Ref		Ref		Ref	
Peripheral blood	1.03 (0.52–2.05)	0.93	0.86 (0.45–1.67)	0.66	1.19 (0.42–3.41)	0.75	1.04 (0.69–1.56)	0.87	0.88 (0.50–1.53)	0.64	1.17 (0.68–2.00)	0.57
ATG treatment												
No	Ref		Ref		Ref		Ref		Ref		Ref	
Yes	0.91 (0.64–1.30)	0.61	0.80 (0.57–1.13)	0.20	0.67 (0.41–1.11)	0.12	0.75 (0.57–0.98)	0.03	0.95 (0.67–1.35)	0.78	0.67 (0.48–0.94)	0.02
Conditioning												
RIC	Ref		Ref		Ref		Ref		Ref		Ref	
MAC	1.02 (0.58–1.79)	0.95	1.17 (0.66–2.08)	0.59	1.11 (0.48–2.54)	0.81	0.84 (0.65–1.08)	0.16	1.23 (0.88–1.73)	0.23	0.86 (0.62–1.19)	0.36
Patient age												
≤60 years	Ref		Ref		Ref		Ref		Ref		Ref	
>60 years	1.50 (1.07–2.11)	0.02	1.32 (0.93–1.89)	0.12	1.23 (0.71–2.12)	0.46	1.44 (1.06–1.85)	0.007	0.78 (0.52–1.15)	0.20	1.87 (1.34–2.61)	<0.001

* Number of events: OS, n = 167; relapse, n = 159; NRM, n = 73. ** Number of events: OS, n = 254; relapse, n = 142; NRM, n = 160. Abbreviations: ATG, anti-thymocyte globulin; HR, hazard ratio; MAC: myeloablative conditioning; RIC: reduced intensity conditioning.

© 2020 by the authors. Licensee MDPI, Basel, Switzerland. This article is an open access article distributed under the terms and conditions of the Creative Commons Attribution (CC BY) license (http://creativecommons.org/licenses/by/4.0/).
